# Wearable Devices and AI-Driven Remote Monitoring in Cardiovascular Medicine: A Narrative Review

**DOI:** 10.7759/cureus.90208

**Published:** 2025-08-16

**Authors:** N Gaoudam, Sai Krishna Sakhamudi, Bobby Kamal, Nipun Addla, Etikala Pravallika Reddy, Madhulika Ambala, Kanchi Lavanya, Elizabeth Caroline Palaparthi, Alekhya Bhattam, Panneerselvam Periasamy, Suresh Babu Sayana, Tambi Medabala

**Affiliations:** 1 Medical Surgical Nursing, Vinayaka Mission's College of Nursing, Vinayaka Mission's Research Foundation (DU), Karaikal, IND; 2 Internal Medicine, Government Medical College and General Hospital, Kothagudem, IND; 3 Physiology, Government Erode Medical College and Hospital, Erode, IND; 4 Medicine, Kamineni Academy of Medical Sciences and Research Centre, Hyderabad, IND; 5 Internal Medicine, Osmania Medical College, Hyderabad, IND; 6 Internal Medicine, Shasta Regional Medical Center - Prime Healthcare, California, USA; 7 Family and Community Medicine, Rajiv Aarogya Sri Health Care Trust, Hyderabad, IND; 8 Physiology, Government Erode Medical College, Perundurai, IND; 9 Pharmacology, Government Medical College and General Hospital, Kothagudem, IND; 10 Exercise Physiology, Sports Authority of India, Netaji Subhas National Institute of Sports, Patiala, IND

**Keywords:** arrhythmia detection, artificial intelligence, cardiovascular monitoring, diagnostic accuracy, digital health, remote patient monitoring, wearable devices

## Abstract

Background: AI-integrated wearable devices (WDs) offer real-time cardiovascular disease (CVD) monitoring with the potential to improve early detection and patient-centred care. However, the breadth, quality, and clinical applicability of supporting evidence remain variable.

Methods: A narrative review was conducted of peer-reviewed original studies, clinical trials, reviews, and regulatory documents published in English from January 2013 to June 2025. A structured Boolean search identified literature on AI-enabled WDs for cardiovascular monitoring. Selection criteria prioritised randomised trials, prospective observational studies, and real-world implementation reports with a clear regulatory context. Studies were narratively analysed for device classification, physiologic parameters measured, AI techniques, clinical validation outcomes, and evidence of patient engagement.

Results: Evidence demonstrates high diagnostic accuracy for arrhythmia detection and promising outcomes in heart failure monitoring, particularly with Food and Drug Administration or Conformité Européenne-approved devices. Nonetheless, most studies were small, short-term, and conducted in controlled settings, limiting generalisability. Key gaps include underrepresentation of diverse populations, lack of interoperability with electronic health records, limited AI explainability, and incomplete cost-effectiveness evaluation. Patient engagement was inconsistently addressed, with most reporting only basic usability or satisfaction measures rather than co-design or adherence optimisation strategies.

Conclusion: AI-integrated WDs show considerable promise in enhancing CVD detection and management but require large-scale pragmatic trials, standardised interoperability protocols, transparent AI model interpretability, and structured patient involvement. Addressing these gaps will be essential for equitable, scalable, and clinically integrated adoption.

## Introduction and background

Burden of cardiovascular disease (CVD)

CVD remains the leading cause of death globally, accounting for an estimated 20.5 million deaths in 2021, representing approximately one-third of all global deaths. This burden is not confined to high-income countries; low- and middle-income nations now bear more than 75% of the global CVD mortality. Among the most prevalent forms of CVD are ischemic heart disease, stroke, heart failure (HF), and arrhythmias, which frequently present with episodic or silent manifestations that elude timely clinical detection. As populations age and the prevalence of risk factors such as hypertension, diabetes, and obesity increases, the cumulative burden of CVD is projected to rise sharply, with an estimated 35.6 million annual deaths by 2050 if current trends continue. This epidemiological transition highlights the urgent need for scalable, cost-effective tools that facilitate early diagnosis, longitudinal monitoring, and preventive interventions at the population level [1].

Traditional models of cardiovascular care, which depend on in-person visits and static diagnostic testing, are increasingly inadequate to address this growing burden. Many cardiac conditions, including paroxysmal atrial fibrillation (AF), nocturnal arrhythmias, and transient blood pressure (BP) surges, occur unpredictably and remain undetected during scheduled clinical encounters. Missed opportunities for early diagnosis not only delay therapeutic intervention but also contribute to downstream complications such as stroke, decompensated HF, and sudden cardiac death. Furthermore, disparities in access to speciality care and diagnostics, particularly in rural, remote, and underserved communities, exacerbate the global inequity in CVD outcomes. Therefore, continuous, remote, and patient-centric monitoring approaches are crucial for shifting the paradigm from reactive disease treatment to proactive cardiovascular risk management [2]. In this context, wearable devices (WDs) integrated with AI-driven analytics offer a transformative solution. They enable the real-time acquisition of physiologic signals in naturalistic environments, empowering both patients and clinicians with timely, actionable insights. Such technologies not only promise to bridge gaps in access and surveillance but also align with broader public health goals of early detection, risk stratification, and personalised intervention. As health systems strive to control CVD morbidity and mortality, continuous cardiovascular monitoring stands out as a pivotal tool in improving outcomes and reducing healthcare costs across diverse populations [3].

Rise of digital health and AI-driven wearable technologies

Parallel to this evolution is the rapid growth of digital health technologies, particularly WDs capable of acquiring real-time, continuous physiologic data outside the clinical setting. Devices such as smartwatches, patches, rings, and textile-based sensors now enable the seamless capture of vital cardiovascular signals, including heart rate (HR), electrocardiogram (ECG), photoplethysmography (PPG), HR variability (HRV), BP, and oxygen saturation (SpO₂). These technologies have transformed consumer-grade wearables into medically viable tools for detecting arrhythmias, managing hypertension, and monitoring HF [4].

AI, particularly machine learning (ML) and deep learning (DL), has emerged as a critical enabler of this digital transformation. AI algorithms can analyse large volumes of wearable data to detect patterns, classify cardiac rhythms, predict adverse events, and support clinical decision-making. For example, AI-powered systems can accurately identify AF episodes from noisy, real-world PPG or ECG data, approaching the accuracy of traditional diagnostics. Furthermore, AI models offer the potential to integrate multimodal data streams, including behavioural, biometric, and contextual information, to create personalised cardiovascular risk profiles and proactive care pathways [5]. This confluence of AI and wearable technology represents a paradigm shift from episodic care to continuous, predictive, and participatory cardiovascular health management. As health systems seek to reduce hospitalisations, improve patient engagement, and extend care into the home and community, AI-integrated wearables are poised to become foundational tools in the future of cardiovascular medicine.

Need for remote monitoring solutions

The COVID-19 pandemic catalysed a dramatic reconfiguration of healthcare delivery worldwide, accelerating the adoption of remote care models and exposing the limitations of traditional in-person monitoring for chronic diseases such as CVD. As lockdowns, resource reallocation, and patient hesitancy restricted access to clinical settings, the need for alternative, decentralised, and continuous monitoring tools became urgently apparent. Patients with CVD, particularly those with HF, arrhythmias, or hypertension, experienced disruptions in follow-up care, leading to increased risk of deterioration and missed therapeutic windows. In response, health systems rapidly pivoted to telehealth, virtual consultations, and digital health platforms to maintain continuity of care. Particularly, wearable cardiovascular devices emerged as a key enabler of this transition, offering non-invasive, real-time physiologic surveillance outside the hospital environment [5-8].

This shift was not merely a temporary workaround but a structural transformation in how cardiovascular care is conceptualised and delivered. Regulatory bodies fast-tracked approvals for remote diagnostic tools, reimbursement models were expanded to include virtual monitoring services, and patients became more receptive to self-tracking technologies. The pandemic thus served as both a stress test and a proving ground for the viability of remote monitoring at scale. Additionally, clinical research accelerated its integration of digital endpoints and decentralised trial designs, further validating the role of AI-powered WDs in clinical and investigational settings. As the global healthcare community adapts to a post-pandemic reality, the momentum behind remote cardiovascular monitoring is not only sustained but increasingly institutionalised, driving long-term investment and innovation in this domain. Several policy shifts during the pandemic, such as emergency use authorisations granted by the U.S. Food and Drug Administration (FDA) and other regulatory bodies, were time-limited responses to crisis needs. However, other reforms, such as the integration of remote monitoring codes into Medicare and Medicaid billing systems (e.g., CMS 99453-99458), and broader acceptance of digital tools in standard care guidelines, have persisted beyond the pandemic, indicating lasting changes in health policy. Distinguishing between these short-term and long-term changes is essential for forecasting the sustainable implementation of AI-driven wearable solutions [7,9,10].

Scope and objectives

This review examines the combined use of WDs and AI to monitor heart health/CVDs remotely. It focuses on how effectively these tools detect heart issues, their usefulness in real-world settings, and real-world utility (refers to the performance, integration, and clinical value of AI-integrated WDs outside of controlled trial settings. Evidence included spans observational studies, pragmatic trials, post-market surveillance, and early-stage implementation research.), and their potential role in everyday medical care. The review details various types of devices and AI technologies, highlighting their advantages and current limitations, and identifies areas that require improvement. The primary objective is to present a narrative overview of the current state of this technology and explore how it can be more effectively utilised to support heart care in the future.

## Review

Database search and screening

This narrative review included peer-reviewed original research articles, clinical trials, review articles, and regulatory documents published between January 2013 and June 2025, focusing specifically on AI-integrated wearable technologies applied to cardiovascular monitoring. The search strategy employed a structured Boolean query: ("wearable devices" OR "smart wearables" OR "wearable sensors" OR "digital health devices") AND ("artificial intelligence" OR "machine learning" OR "deep learning" OR "AI") AND ("cardiovascular disease" OR "heart disease" OR "cardiac monitoring" OR "cardiac arrhythmia" OR "atrial fibrillation") AND ("remote monitoring" OR "telemonitoring" OR "remote patient monitoring") AND ("diagnostic accuracy" OR "clinical utility" OR "real-world implementation" OR "regulatory challenges"). Only English-language publications were considered. Exclusion criteria included duplicate records, non-English articles, grey literature (such as preprints, editorials, and conference abstracts), and studies lacking relevance to cardiovascular applications or sufficient technical validation data (e.g., missing performance metrics or regulatory context).

Although no formal quality scoring system was applied, each study was appraised based on methodological design, sample size, presence of clinical endpoints, and evidence of regulatory status (e.g., FDA or Conformité Européenne (CE) approval). Greater weight was given to randomised trials, prospective observational studies, and real-world deployment data. The extent and clarity of AI integration were also considered when interpreting relevance. Potential publication and commercialisation bias, particularly in device-focused studies backed by industry, were acknowledged. Studies with independent validation or publicly available evaluation protocols were prioritised where applicable.

Classification of WDs in cardiovascular medicine

Physiological Parameters Captured

WDs in cardiovascular medicine are designed to monitor a spectrum of critical physiological parameters. Foremost, among these is the ECG, with single-lead or multi-lead configurations embedded in smartwatches, chest patches, and novel e-skin platforms enabling both real-time and retrospective rhythm analysis. Accuracy for AF detection can reach 98.3% sensitivity and 99.6% specificity in FDA-cleared devices such as the Apple Watch (Apple Inc., Cupertino, California) [11,12]. PPG technology, employing optical sensors, highlights HR, HRV, SpO₂, and BP estimation in wristbands, rings, and garment-integrated sensors; recent AI-enhanced preprocessing (e.g., CycleGAN, RLS adaptive filtering) has reduced motion artefacts by up to 49% [13,14]. HRV remains a valuable prognostic marker for arrhythmogenic disorders and HF. Non-invasive BP estimation, leveraging pulse transit time, bioimpedance, capacitive sensing, and oscillometric surrogates, has shown clinically acceptable error margins, with graphene electronic tattoos achieving ±4.5 mmHg (diastolic BP, DBP) and ±5.8 mmHg (systolic BP, SBP) in line with the Institute of Electrical and Electronics Engineers (IEEE)-Grade A performance [15], and bioimpedance smart rings reporting DBP error of 0.11±3.87 mmHg and SBP error of 0.11±5.27 mmHg [11]. Advanced platforms also integrate red/infrared spectroscopy for continuous SpO₂ monitoring, where prototype arterial SpO₂ sensors achieved root mean square error (RMSE) of 2.1% compared to gold-standard oximetry, aiding HF and sleep apnoea management [11-15].

Implantable Cardiovascular Devices

Traditionally, implantable devices such as pacemakers and implantable cardioverter-defibrillators (ICDs) have played a central role in managing cardiac rhythm disorders. Pacemakers restore normal heart rhythm in patients with bradycardia or tachycardia by delivering electrical impulses, while ICDs are equipped to detect and terminate life-threatening arrhythmias through internal shocks. Although larger due to battery requirements, ICDs offer expanded functionality beyond pacing. Pacemakers and ICDs remain central to rhythm management, with modern systems miniaturised, wireless, and magnetic resonance imaging-compatible, yet still constrained by procedural risks, battery life, and cost (>USD 20,000 in some cases) [16]. Insertable cardiac monitors (ICMs) such as the Reveal LINQ offer long-term ECG storage for arrhythmia diagnosis, with 79.5% daily wireless transmission success and detection of multiple arrhythmic events in first-in-human trials [17]. The FDA and CE-approved CardioMEMS™ system enables daily pulmonary artery pressure monitoring in HF, reducing hospitalisations by up to 84% in observational data [18]. AI integration across implants supports automated detection (diagnosis) and predictive risk scoring (e.g., HF events with 76%-88% sensitivity, 85% specificity in ICM/CardioMEMS data) [11]. Limitations span invasiveness, occasional migration or signal loss, and access inequity [11,16-18].

ECG Monitoring Devices

Monitoring is fundamental for detecting cardiac arrhythmias and evaluating heart function. Traditional 12-lead ECGs, though accurate, are non-portable and limited to clinical settings. Wearable technologies now offer portable alternatives for continuous ECG tracking. Holter monitors, as body-worn devices, enable multi-lead ECG monitoring for 24 hours or more, facilitating arrhythmia diagnosis. Holter monitors provide multi-lead, 24-48-hour ambulatory data, while newer WDs enable longer, more comfortable wear. Savvy personal mobile ECG sensor reduced unnecessary referrals in primary care by 64% without loss of diagnostic yield [19]. Apple Watch single-lead ECG yields up to 100% sensitivity for AF when manually reviewed [12], with automated algorithms achieving 81.1% sensitivity and 99.5% specificity. Emerging e-skin wearables (e.g., MXene-PU mesh) deliver >99% arrhythmia classification accuracy using convolutional neural network-long short-term memory (CNN-LSTM) models [20]. Limitations include motion artefacts, single-lead constraints, and skin-contact variability; AI primarily supports diagnosis and noise removal [12,19,20].

HR Monitoring Devices

HR is a key cardiovascular metric that varies with physiological and pathological states. While normal resting HR ranges from 60-100 bpm, it may rise in conditions such as hypertension or HF. HR can be measured through two primary modalities: electrical and PPG. (1) Electrical-based wearables: ECG-based wearables detect HR using R-R interval algorithms such as Pan-Tompkins. (2) PPG-based wearables: Commercial devices such as Apple Watch and Fitbit (Fitbit LLC, San Francisco, California, USA) use PPG, which leverages light absorption to assess blood volume changes. Red and green light-emitting diodes are common, with green preferred due to lower motion sensitivity. PPG data are often processed using peak detection algorithms but are vulnerable to motion artefacts. Electrical-based wearables, such as piezoelectric chest sensors, detect HR within ±1 bpm of reference devices with >95% abnormality detection accuracy [21]. PPG-based consumer wearables benefit from accelerometer and electromyography (EMG) integration; EMG-assisted artefact removal can reduce HR estimation error by 49% [13]. Remote PPG via smartphone cameras achieved a mean absolute error of 3.58±2.4 bpm, expanding low-cost, non-contact options [22]. Comfort, sensor placement, and skin tone remain important adherence and accuracy determinants [13,21,22].

BP Monitoring Devices

BP is a key cardiovascular parameter that reflects the force of blood against arterial walls and is typically measured as systolic (during heart contraction) and diastolic (during heart relaxation) pressure. Recent advances have led to the development of cuffless wearable BP monitors that enable non-invasive, continuous tracking using techniques such as PPG, bioimpedance, and capacitive pressure sensing. Aktiia’s CE-marked cuffless PPG monitor matches ambulatory BP trends but requires monthly calibration [11]. Bioimpedance rings and graphene tattoos show near-ISO 81060-2 performance, and capacitive polydimethylsiloxane-polyethylene terephthalate sensors detect oscillometric waveforms across the physiological BP range [23]. AI use spans preprocessing (noise removal) to regression-based BP estimation (diagnosis). Limitations include motion sensitivity, calibration dependence, and reduced accuracy after exercise [11,23].

Thoracic Fluid Index (TFI)

The TFI is a critical parameter for assessing intrathoracic fluid status, particularly in the management of HF. With an estimated global burden of over 64 million patients with HF, vigilant fluid monitoring is essential for early detection of decompensation and optimising therapy. Traditionally, TFI is measured using implantable devices such as ICDs and CardioMEMS™, but recent advancements have enabled non-invasive monitoring through wearable technologies. The CoVa™ system (a necklace-shaped device) detected >20% TFI changes with 100% sensitivity up to five days before HF events in small cohorts [24]. Similarly, µCor monitors report 38% hospitalisation reductions in patients with HF. Limitations involve bioimpedance variability, electrode placement consistency, and lack of BP data. AI trend analysis supports both diagnosis and early prediction [24].

Smart Rings and Garment-Based Biotextiles

Smart rings and garment-based biotextiles are emerging as wearable technologies in cardiovascular monitoring. Smart rings, such as the Oura Ring®, offer reliable HR tracking with minimal bias (0.4 bpm), are user-friendly, and have long-term wearability, though sleep phase accuracy remains variable across studies. Garment-based biotextiles integrate biosensors into clothing to enable continuous, high-fidelity ECG and respiratory monitoring, with signal quality approaching that of Holter monitors. Meta-analysis shows smart rings achieve ECG-based HR tracking with a mean bias of -0.4 bpm and AF detection accuracy of 96.9% (sensitivity: 98.9%, specificity: 94.3%, and area under the curve: 0.99) [25]. Cuffless BP via Senbiosys® yielded SBP bias of 2.3±11.3 mmHg (RMSE=7.3) and DBP bias of 0.5±6.9 mmHg (RMSE=3.6). Limitations include motion artefacts, optical skin-tone bias, BP estimation variability, and interoperability gaps. Garment-based systems enable Holter-quality ECG and respiratory monitoring but require robust washability and electrode placement consistency [25]. Key findings of WDs are summarised in Table 1.

**Table 1:** Summary of key studies on AI-integrated and conventional wearable/clinical cardiovascular monitoring devices. AI: Artificial Intelligence; AF: Atrial Fibrillation; AV: Atrioventricular; BP: Blood Pressure; CHD: Coronary Heart Disease; CNN: Convolutional Neural Network; CO: Cardiac Output; CPET: Cardiopulmonary Exercise Testing; DBP: Diastolic Blood Pressure; ECG: Electrocardiogram; EE: Energy Expenditure; EMD: Empirical Mode Decomposition; EMG: Electromyography; EMD (in CHD context): Electromechanical Delay; FDA: U.S. Food and Drug Administration; HF: Heart Failure; HGCP: Handgrip Contraction Protocol; HR: Heart Rate; HRV: Heart Rate Variability; HUT: Head-Up Tilt; ICC: Intraclass Correlation Coefficient; ICM: Insertable Cardiac Monitor; IoT: Internet of Things; LoA: Limits of Agreement; LSTM: Long Short-Term Memory; LVET: Left Ventricular Ejection Time; MAE: Mean Absolute Error; MAPE: Mean Absolute Percentage Error; MPM: MXene-PU Mesh; NIBP: Non-Invasive Blood Pressure; PCG: Phonocardiogram; PEP: Pre-Ejection Period; PF: PhysioFlow; P-SENSE: P-Wave Based Sensing Algorithm; PPG: Photoplethysmography; PVCs: Premature Ventricular Contractions; PVDF: Polyvinylidene Fluoride; rPPG: Remote Photoplethysmography; RLS: Recursive Least Squares; SNR: Signal-to-Noise Ratio; SV: Stroke Volume; USCOM: Ultrasonic Cardiac Output Monitor; Valsalva: Valsalva Maneuver.

Author(s) (Year)	Sample Size	Study Design	AI Use	Device Type (Consumer vs. Clinical)	Regulatory Approval Status	Key Findings	Patient-Reported Outcomes	Limitations	Evidence Gaps
Pürerfellner et al. (2015) [17]	30 patients	Prospective, multicenter, non-randomised, single-arm clinical usability study	No AI used	Clinical (ICM Reveal LINQ)	FDA marked at the time of the study	Miniaturised Reveal LINQ ICM showed high R-wave sensing performance, 79.5% daily wireless transmission success, ease of implantation (90% rated easy/very easy), no major complications and detected multiple arrhythmia types within 1 month; minimal migration and good ECG quality	96.7% rated the home monitor as very easy to use; 76.7% “very satisfied” and 20% “satisfied” with the device; no limitation in daily activities reported	Short follow-up (1 month), small sample size, no adjudication of arrhythmia detection accuracy in this study and reliance on ventricular ECG for AF detection	Long-term safety and performance data, comparative accuracy studies with other monitoring systems, evaluation of AF detection with P-SENSE algorithm in larger cohorts, and real-world cost-effectiveness data
Zang et al. (2025) [26]	40 cases (20 healthy and 20 patients with coronary heart disease)	Experimental device development, bench testing, and human volunteer validation	Deep learning algorithms planned for future classification/prediction	Wearable (clinical-grade prototype) integrating ECG and PCG	Not yet approved/regulatory status not mentioned	Developed a non-invasive wearable integrating PZT-based PCG and ECG acquisition with high SNR; demonstrated stable signal quality under varying conditions; extracted joint cardiac parameters (EMD, LVET, and PEP) differentiating healthy patients and patients with CHD	Not reported	Small sample size; tested mainly in controlled settings; limited patient diversity; no long-term monitoring data; AI-based classification not yet implemented	Lack of large-scale clinical trials; no regulatory pathway defined; absence of patient usability and comfort studies; need for validation in real-world ambulatory scenarios
Vodička et al. (2021) [19]	400 patients (plus 30 family physicians)	Observational, prospective, randomised, cohort study	No explicit AI use reported	Consumer-grade personal mobile ECG sensor (Savvy) used in a clinical setting	Not specified	Use of personal ECG sensor in primary care significantly reduced referrals to cardiologists (11.5% vs. 32% in control) without compromising detection of rhythm disorders; facilitated timely treatment and reduced unnecessary healthcare costs	82%–98% of patients found it easy to install/use, were not bothered by the sensor, gladly wore it, and had no usage problems.	Low number of male participants; incomplete data for some patients/physicians; lack of blinding in physician intervention choice; physician reluctance to participate	Need for more well-designed interventional studies at the primary care level using telecardiology devices; long-term clinical and cost-effectiveness outcomes have not been assessed
Alnasser et al. (2023) [12]	469	Cross-sectional comparative study in outpatient cardiac clinics	Automated arrhythmia detection (Apple Watch algorithm) + manual physician interpretation	Consumer (Apple Watch Series 6)	FDA-approved for AF detection	Apple Watch ECG showed high correlation with 12-lead ECG for rhythm, P-wave, PR interval, QRS width, and HR; sensitivity/specificity for AF: 100%/99.18% (manual), 81.08%/99.54% (automated); capable of detecting first-degree AV block, PVCs, and wide QRS	Not assessed directly in the study	Self-reported medical history may cause bias; ECG interpretation by junior physicians, conducted only in cardiac patients; single-lead ECG limitations	Need for larger studies, including broader populations; evaluation of patient usability and adherence; performance for ischaemia/ST changes in multi-lead smartwatch ECGs; validation of detection for non-AF abnormalities
Cui et al. (2023) [20]	Not specified (material/device development study)	Experimental, development, and validation study of MPM electronic skin integrated with AI-enabled ECG monitoring	Hybrid CNN and LSTM algorithm for arrhythmia detection	Clinical-grade wearable 12-lead ECG e-skin system	Not yet regulatory-approved (prototype research stage)	Developed breathable, conformal MPM e-skin with double-layer conductive network, enabling long-term (≥7 days) comfortable wear, ultra-low impedance (4.68 kΩ at 1 kHz), high SNR (16.5 dB), and multifunctional sensing. Integrated system achieved >99% diagnostic accuracy for four arrhythmias during 12 hours of daily use. Also detects EMG, subtle vibrations, and large joint motions.	Not directly reported (patient comfort inferred from high breathability and skin compatibility)	No human clinical trial with diverse population; regulatory pathway and mass manufacturing not addressed	Lack of large-scale clinical validation; absence of longitudinal patient adherence data; real-world performance in varied environments untested
Ji and Zhang (2022) [27]	Not specified (device prototyping and lab validation study)	Experimental design, fabrication, and bench/volunteer validation of a highly sensitive stretchable piezoelectric strain sensor for respiratory and heartbeat monitoring	EMD algorithm in MATLAB for signal separation	Consumer-grade wearable sensor prototype	Not approved/regulatory status not mentioned	Developed a serpentine-layout PVDF-based piezoelectric strain sensor (thickness: 168 µm, voltage sensitivity: 0.97 mV/με, and modulus: 13.5 MPa), enabling real-time respiratory and heartbeat monitoring; successfully separated signals via EMD; potential use for COVID-19 self-diagnosis	Not reported	Prototype-stage testing only, small-scale validation, motion artefacts during activity, output differences between simulation and experiment	Large-scale clinical validation absent; no long-term durability data; no regulatory pathway explored; no patient usability/adherence assessment
Mokhtari et al. (2019) [21]	10 human participants	Prototype development and validation study	Correlation-based algorithm for abnormality detection	Clinical (wearable Bluetooth-enabled piezoelectric chest sensor)	Not specified	Developed a portable, Bluetooth-enabled piezoelectric sensor system to measure HR, beat period, and pulse pressure; validated against conventional metres (≤3% error); correlation-based algorithm achieved >95% accuracy in detecting seven types of cardiac abnormalities	Reported ease of use, comfort, and minimal interference with daily activities	Small sample size; breath-hold condition during measurements; prototype stage without large-scale clinical validation	Lack of regulatory clearance data; no long-term performance assessment; limited testing in real-world conditions; absence of diverse patient populations, including those with known cardiac diseases
Falter et al. (2019) [28]	40 patients with cardiovascular disease	Cross-sectional validation study during CPET	None	Consumer (Apple Watch Sport 42 mm, 1st gen)	Not FDA/CE approved as a medical device	Apple Watch measured HR with clinically acceptable accuracy (ICC: 0.729–0.958; MAPE: 6.33%–10.69%) during exercise; no systematic HR error; systematically overestimated EE (MAPE: 114.72%; bias: +30.47 kcal). HR accuracy highest at maximal intensity; EE accuracy poor	Not assessed	Single-center, non-randomised; heterogeneous cardiac diagnoses; male predominance; no arrhythmia subgroup; EE algorithm undisclosed; no assessment of skin tone/BMI effects; CPET only, no post-exercise readings	Lack of subgroup analyses (e.g., arrhythmia and diverse skin tones/BMI); no evaluation in free-living conditions; no assessment of long-term monitoring reliability; need for manufacturer transparency in EE algorithms
Wen et al. (2023) [14]	Dataset I: public dataset; Dataset II: 12 volunteers (9 males and 3 females; age 22–36 years)	Experimental evaluation with two datasets – algorithm validation (Dataset I) and real-time wearable testing in natural settings (Dataset II)	Decision tree for heart-rate spectral interval classification; multisensor fusion RLS adaptive filtering for motion artefact removal	Consumer wearable prototype (wrist-worn PPG + accelerometer, IoT-enabled)	Not stated; research prototype without formal regulatory clearance	Developed low-order RLS adaptive filter with decision tree-based spectrum selection to improve HR estimation accuracy during motion; achieved AAEP ≈10.68% compared to higher errors in LMS/NLMS; processing time per second ≈2.15 ms, enabling real-time operation	Not assessed	Small sample size in real-world testing; limited to short-duration protocols (16 min); tested only on healthy young adults; performance vs. gold-standard ECG in broader populations not established	No clinical validation in diverse patient groups; no assessment under pathological conditions or extreme motion; long-term durability and usability data lacking; regulatory pathway not addressed
Friman et al. (2022) [13]	9	Experimental study with within-subject comparison across movement tasks	No AI used; signal processing via spectrum subtraction algorithms	Consumer (smartwatch prototype)	Not specified (prototype; no regulatory clearance)	Adding wrist surface EMG to accelerometer-based artefact removal in PPG smartwatches reduced HR estimation MAE by 49%, improved robustness by lowering variability across subjects, and enhanced accuracy during activities with tissue movement	Not reported	Small sample size; adhesive electrodes used (impractical for real-world smartwatch); no anthropometric/fitness data; algorithm ignores HR<48 BPM; limited to frequency-domain approach; no irregular MA handling	Need for larger, diverse cohorts; feasibility without adhesive electrodes; handling irregular motion artefacts; improving low HR estimation; real-world validation in varied populations
Hosni and Atef (2023) [22]	7 subjects (3 men, 2 women, and 2 children)	Experimental validation study using contactless smartphone-based rPPG HR measurement	No explicit AI; algorithm-based signal processing (Mexican hat wavelet, recursive baseline-wander removal, adaptive FFT peak detection)	Consumer (Samsung S20 smartphone camera)	Reference validation with FDA-approved BEURER PO30 pulse oximeter; proposed system itself not reported as regulatory-approved	Proposed algorithm achieved real-time, contactless HR measurement from 0.4 m without extra sensors; MAE ± SD = 3.58±2.4; improved worst-case peak detection accuracy by >37.5%; robust against motion artifacts via adaptive wavelet filtering and recursive normalisation	Not assessed	Accuracy reduced with distance >0.4 m; performance influenced by background light; tested only indoors under fixed lighting; small sample size; no diverse skin tone evaluation	Need validation in larger, more diverse populations; performance under variable lighting and outdoor conditions; regulatory clearance pathway not addressed; integration into consumer-ready application not yet implemented
Kireev et al. (2022) [15]	N=7 healthy adults (mid-20s)	Proof-of-concept experimental study with multiple BP elevation manoeuvres (HGCP, Valsalva, cycling)	Machine learning regression (AdaBoost) to map Bio-Z waveform features to BP values	Wearable graphene electronic tattoos (clinical research prototype)	Not yet approved; proof-of-concept stage	Demonstrated continuous cuffless BP monitoring for >300 minutes using graphene bioimpedance tattoos with Grade A accuracy (0.2±4.5 mmHg DBP, 0.2±5.8 mmHg SBP); superior performance vs. Ag wristband; durable under motion, sweat, and water immersion; enabled respiration rate estimation	No adverse skin reactions, irritation, or discomfort reported during prolonged wear	Small, homogeneous healthy sample; no patients with hypertension; short-term lab-based validation; requires initial calibration; accuracy reduced post-workout and after days without recalibration	Need for large-scale, long-term ambulatory trials; validation in diverse populations, including patients with hypertension; regulatory pathway definition; integration into consumer-grade wearable platforms
Bijender and Kumar (2021) [23]	Not applicable (device development and lab validation study)	Experimental device fabrication and performance evaluation	None	Clinical (wearable BP monitoring sensor prototype)	Not approved/regulatory status not mentioned	Developed a flexible capacitive pressure sensor with porous PDMS-DIW dielectric layer showing high sensitivity (up to 0.095 kPa⁻¹ in 100–500 Pa range), ultra-low detection limit (1 Pa), fast response/recovery (~110 ms), and linear BP detection in 55–220 mmHg range; capable of capturing oscillometric waveforms for different BP states	Not assessed (no human patient trials; tested on NIBP analyzer with artificial arm mandrel)	Sensitivity improvement still needed for better oscillometric waveform resolution; no real-world patient data; limited to lab simulations	Lack of clinical validation in human subjects; no long-term stability or usability data; absence of AI-based signal processing; regulatory pathway and integration with existing BP devices not explored
Cheung et al. (2020) [29]	20 healthy, well-trained university athletes	Comparative experimental study using HUT test; PF vs. USCOM	None	Consumer-oriented portable cardiac output monitor (PhysioFlow PF07 Enduro) compared with clinical Doppler CO monitor (USCOM)	Not stated	PhysioFlow accurately measured HR but overestimated stroke volume (positive bias: 62%–94%) and underestimated relative SV change (negative bias: −42% to −80%); CO readings were anomalous at high tilt; accuracy deteriorated at 70° suggesting gravitational influence; suitability for sports use questionable	No adverse events; no discomfort or interference reported	Limited to HUT test (restricted physiological range); no gold-standard invasive reference; possible gravitational influence unexplained; findings may not generalise to full sports intensity	Lack of validation in broader sports settings; absence of calibration correction; untested in “sports range” physiological extremes; unclear algorithmic limitations in young athletic adults
Khandwalla et al. (2016) [24]	20 (18 completed)	Pilot observational study	Algorithmic analysis of hemodynamic data for event prediction	Clinical (wearable CoVa™ Monitoring System)	Not stated	Daily monitoring of stroke volume and thoracic fluid index predicted HF hospitalisations and other cardiac events; vital signs and weight were not predictive	Well tolerated; high compliance (~75%)	Small sample size; short follow-up; only pilot data; 2 dropouts	Need larger-scale validation; unclear regulatory clearance; generalisability to broader HF population untested

AI techniques and algorithms in wearable cardiovascular monitoring

The convergence of WDs and AI has ushered in a new era of cardiovascular healthcare, one defined by real-time monitoring, predictive diagnostics, and personalised treatment. At the heart of this transformation lies a sophisticated ecosystem of ML and DL models, advanced signal processing pipelines, and interpretability frameworks that collectively empower clinicians with actionable insights from continuous physiological data.

Commonly Used AI Models

AI in wearable cardiology spans a spectrum from traditional ML algorithms to advanced DL frameworks. Classical models such as support vector machines, random forests, k-nearest neighbours, and gradient boosting decision trees have been widely adopted for arrhythmia classification, BP estimation, and ECG-based diagnostics. These rely on handcrafted features derived from HRV, frequency spectra, or ECG morphology. In contrast, DL models such as CNNs and LSTMs automatically learn spatial and temporal patterns directly from raw ECG or PPG signals, enabling superior performance in complex scenarios such as multi-class arrhythmia detection. Recent studies have highlighted the potential of hybrid architectures, such as CNN-LSTM and attention-based transformers, which simultaneously capture spatial hierarchies and long-range dependencies. These are particularly beneficial in dynamic cardiac conditions where signal morphology varies over time. Lightweight models, such as temporal convolutional networks (TCNs), are also gaining prominence for their efficiency and suitability for edge deployment on wearable hardware [30].

Signal Processing and Feature Engineering

Wearables are prone to motion artefacts, baseline drift, and ambient noise, especially in ambulatory settings. Advanced denoising methods, such as denoising autoencoders, empirical mode decomposition, and cycle-consistent generative adversarial networks (CycleGANs), are employed to reconstruct clean signals from noisy input. Zargari et al. demonstrated that CycleGANs can denoise PPG signals up to 9.5 times better than accelerometer-based correction, with a 45% improvement in energy efficiency. Feature extraction remains critical in traditional pipelines. Parameters such as RR intervals, QRS duration, spectral entropy, and P-wave asymmetry are routinely engineered to detect anomalies such as AF or ischemic trends with high precision [31].

Explainability and Model Interpretability

Despite their success, DL models must further gain the clinician's trust. Explainable AI (XAI) techniques such as SHapley Additive exPlanations (SHAP) and Local Interpretable Model-Agnostic Explanations (LIME) address this by visualising the most influential features in a prediction. For example, AI-predicted anomalies in RR intervals or ST segments can be mapped back to ECG segments for physician review. In electrophysiology, XAI has informed ablation planning and optimised device follow-ups. Regulatory bodies are increasingly requiring explainability as part of AI validation pipelines, accelerating its integration into clinical software [32].

Translational Advances and Future Trends

Beyond prediction, AI is transforming cardiovascular monitoring into a proactive paradigm. Applications now span echocardiography (e.g., EchoGo for automated left ventricular ejection fraction/strain prediction), signal-derived age prediction, and federated learning for privacy-preserving cross-hospital model training. Studies using AI on echocardiographic strain and cardiac time intervals (e.g., Hemotag) have demonstrated promise in detecting decompensated HF. The field is rapidly moving toward multimodal, multi-sensor fusion models that integrate wearables, electronic health records (EHRs), imaging, and genomics for holistic cardiovascular profiling. Together, these innovations are not just enhancing diagnostic accuracy but redefining the frontiers of precision cardiology. The evolving AI-wearable ecosystem holds transformative potential for both acute detection and long-term disease management [33].

Clinical validation and real-world applications (clinically grounded and connected to practical implementation)

Clinical validation of AI-enabled WDs in cardiovascular medicine extends beyond demonstrating statistical accuracy in controlled environments; it requires confirmation of analytical validity, clinical validity, and, most importantly, clinical utility in routine care [32]. For arrhythmia detection, particularly AF, multiple large-scale trials and meta-analyses have established that single-lead smartwatch ECGs and continuous PPG-based monitoring can achieve sensitivities and specificities exceeding 95% when benchmarked against 12-lead ECG, provided that rhythm-strip interpretation is confirmed by a clinician [12]. Particularly, the Apple Heart Study demonstrated the feasibility of population-level AF screening via irregular pulse notifications, while updated American College of Cardiology/American Heart Association/American College of Clinical Pharmacy/Heart Rhythm Society guidelines now acknowledge the role of ambulatory technologies in AF detection, with the critical stipulation that diagnosis requires ≥30 seconds of documented ECG reviewed by a healthcare provider [12]. Smart rings, such as the Oura Ring®, have shown comparable HR accuracy to wrist-worn devices and, when coupled with DL algorithms, deliver AF detection sensitivities of nearly 99% [25], although their ability to classify non-AF arrhythmias remains limited. In primary care, pragmatic use of mobile ECG sensors such as SAVVY has reduced unnecessary cardiology referrals by more than half without compromising diagnostic yield, illustrating real-world cost-effectiveness when incorporated into structured triage workflows [19].

In HF management, implantable hemodynamic monitors such as the CardioMEMS™ system have demonstrated reductions in HF hospitalisations of up to 33% in randomised trials and consistent benefits in real-world registry data when integrated into nurse-physician-led remote monitoring programs [18]. Optimal use requires daily pulmonary artery pressure review, predefined thresholds for alerts, and rapid therapy adjustments, with outcome metrics, including hospitalisation rates, natriuretic peptide changes, and patient-reported quality-of-life scores [11]. Non-invasive thoracic fluid monitoring solutions, such as the CoVa™ system, have also shown promise in pilot studies for predicting decompensation several days in advance [24], though larger, outcome-focused trials are still needed before widespread adoption. In hypertension care, cuffless BP devices ranging from PPG-based smart rings to graphene electronic tattoos have achieved mean biases within ±5 mmHg compared to invasive or auscultatory standards in controlled settings [15]. However, calibration dependence, motion sensitivity, and post-exercise drift necessitate confirmatory use of validated cuffs before therapeutic decisions, aligning with current device labelling and best practice recommendations [11].

From a workflow perspective, clinical impact is maximised when device selection is matched to patient risk profiles, spot ECGs for symptomatic AF suspicion, continuous PPG for opportunistic screening, implantable monitors for cryptogenic stroke or unexplained syncope, and hemodynamic sensors for high-risk patients with HF [17,19]. Integration into the EHR through interoperable standards such as HL7 FHIR enables automated alerting, structured data review, and closed-loop care pathways [2]. Reimbursement models, such as U.S. Medicare’s CPT codes 99453-99458 for remote physiologic monitoring, have facilitated sustainable adoption by compensating both setup and ongoing data review, provided regulatory documentation and minimum data-day thresholds are met [7]. Crucially, real-world deployment must address equity and usability through multilingual patient education, simplified onboarding, accessible form factors for older adults, and clinician verification to avoid over-reliance on automated outputs. When implemented with these safeguards, AI-integrated wearables can shift cardiovascular care from episodic and reactive to continuous, proactive, and patient-centred, delivering meaningful reductions in delayed diagnoses, preventable hospitalisations, and overall care costs [3].

Research gaps and future directions

Despite substantial progress in AI-integrated wearable cardiovascular monitoring, several gaps hinder translation from promising prototypes to universally adopted clinical tools. First, most validation studies remain small-scale, short-duration, and conducted in controlled settings, limiting generalisability to diverse patient populations and real-world conditions [19,15]. Large, multicenter pragmatic trials are needed to evaluate device performance across varying comorbidities, skin tones, activity levels, and environmental conditions, with clear linkage to hard outcomes such as mortality, hospitalisation rates, and cost savings. Second, device accuracy often deteriorates under motion, poor skin contact, or suboptimal lighting for optical sensors; robust AI-based artefact correction pipelines, benchmarked against clinically accepted error thresholds, must be prioritised for regulatory approval. Third, interoperability remains inconsistent; few devices achieve seamless HL7 FHIR-compliant integration with EHRs, limiting automated triage, longitudinal tracking, and closed-loop care pathways. Establishing mandatory interoperability standards and embedding AI outputs into clinician-facing dashboards will be pivotal for uptake.

From a regulatory perspective, pathway clarity is lacking for emerging devices such as graphene electronic tattoos and hybrid multimodal platforms, particularly in defining safety, calibration stability, and long-term wear tolerability. Collaborative frameworks between device developers, regulators, and clinical societies are needed to accelerate clearance without compromising patient safety. Reimbursement remains another bottleneck, while CPT codes (e.g., 99453-99458) have enabled adoption in the U.S., similar policy scaffolds are absent in many low- and middle-income countries where CVD burden is greatest. Targeted health-economic analyses in these contexts could strengthen the case for payer coverage. Furthermore, explainability and clinician trust in AI predictions remain underdeveloped; embedding interpretable AI tools such as SHAP or LIME into device software, coupled with structured clinician training, could enhance diagnostic confidence and medicolegal defensibility.

Future development should prioritise multimodal sensor fusion, integrating ECG, PPG, hemodynamic, and activity metrics to improve diagnostic accuracy and reduce false positives, especially for conditions such as paroxysmal AF and HF decompensation. Continuous post-market surveillance with publicly accessible performance registries can detect algorithm drift and demographic bias early. Equally, user-centred design is critical: form factors must cater to older adults, patients with limited dexterity, and underserved populations through simplified interfaces, multilingual support, and low-cost manufacturing. Ultimately, bridging these gaps will require an ecosystem approach combining robust real-world evidence generation, policy alignment, interoperability mandates, and equitable distribution strategies to enable AI-driven wearables to deliver on their potential as scalable, proactive, and cost-effective cardiovascular care solutions.

## Conclusions

AI-integrated WDs have emerged as transformative tools in cardiovascular medicine, offering continuous, patient-centred monitoring that can bridge gaps in detection, risk stratification, and timely intervention. Evidence from both controlled trials and early real-world deployments demonstrates high diagnostic accuracy for arrhythmia detection, clinically meaningful reductions in HF hospitalisations, and potential for cost savings when embedded within structured care pathways. However, the promise of these technologies will only be fully realised through deliberate alignment of research, policy, and practice. Future efforts should focus on conducting large-scale, multicentre pragmatic trials that evaluate device performance across diverse demographics, comorbidities, and environmental conditions, with direct measurement of hard clinical outcomes and health-economic impact. Regulatory pathways must evolve to address novel modalities such as graphene-based sensors and multimodal fusion platforms by setting clear performance, safety, and interoperability benchmarks. Mandatory HL7 FHIR-compliant integration with EHRs, coupled with explainable AI outputs, will be essential to ensure seamless clinician adoption and closed-loop care delivery.

From a policy standpoint, sustained reimbursement mechanisms similar to U.S. CPT codes should be adapted globally, particularly in low and middle-income countries where the CVD burden is highest. Parallel investment in user-centred design, multilingual patient engagement, and low-cost manufacturing can enhance equity and adherence. Finally, continuous post-market surveillance through publicly accessible registries should monitor algorithm performance, bias, and safety in real-world use. By embedding these evidence-based, implementation-oriented strategies into both regulatory and clinical frameworks, AI-driven wearables can transition from promising adjuncts to core components of proactive, scalable, and equitable cardiovascular care.
